# The growth of acronyms in the scientific literature

**DOI:** 10.7554/eLife.60080

**Published:** 2020-07-23

**Authors:** Adrian Barnett, Zoe Doubleday

**Affiliations:** 1 School of Public Health and Social Work, Queensland University of TechnologyBrisbaneAustralia; 2 Future Industries Institute, University of South AustraliaAdelaideAustralia; eLifeUnited Kingdom; eLifeUnited Kingdom

**Keywords:** meta-research, scientific writing, acronyms, communication, knowledge, scientific publishing, None

## Abstract

Some acronyms are useful and are widely understood, but many of the acronyms used in scientific papers hinder understanding and contribute to the increasing fragmentation of science. Here we report the results of an analysis of more than 24 million article titles and 18 million article abstracts published between 1950 and 2019. There was at least one acronym in 19% of the titles and 73% of the abstracts. Acronym use has also increased over time, but the re-use of acronyms has declined. We found that from more than one million unique acronyms in our data, just over 2,000 (0.2%) were used regularly, and most acronyms (79%) appeared fewer than 10 times. Acronyms are not the biggest current problem in science communication, but reducing their use is a simple change that would help readers and potentially increase the value of science.

## Introduction

As the number of scientific papers published every year continues to grow, individual papers are also becoming increasingly specialised and complex (Delanty, 1998; Bornmann and Mutz, 2015; Doubleday and Connell, 2017; Cordero et al., 2016; Plavén-Sigray et al., 2017). This information overload is driving a ‘knowledge-ignorance paradox’ whereby information increases but knowledge that can be put to good use does not (Jeschke et al., 2019). Writing scientific papers that are clearer to read could help to close this gap and increase the usefulness of scientific research (Freeling et al., 2019; Letchford et al., 2015; Heard, 2014; Glasziou et al., 2014).

One feature that can make scientific papers difficult to read is the widespread use of acronyms (Sword, 2012; Pinker, 2015; Hales et al., 2017; Lowe, 2019), and many researchers have given examples of the overuse of acronyms, and highlighted the ambiguities, misunderstandings and inefficiencies they cause (Fred and Cheng, 2003; Narod et al., 2016; Patel and Rashid, 2009; Pottegård et al., 2014; Weale et al., 2018; Parvaiz et al., 2006; Chang et al., 2002). For example, the acronym UA has 18 different meanings in medicine (Lang, 2019). Box 1 contains four sentences from published papers that show how acronyms can hinder understanding.

Box 1. Examples of sentences with multiple acronyms.

The four sentences below are taken from abstracts published since 2000, and reflect the increasing complexity and specialisation of science.

- "Applying PROBAST showed that ADO, B-AE-D, B-AE-D-C, extended ADO, updated ADO, updated BODE, and a model developed by Bertens et al were derived in studies assessed as being at low risk of bias.’ (2019)
- "Toward this goal, the CNNT, the CRN, and the CNSW will each propose programs to the NKF for improving the knowledge and skills of the professionals within these councils.’ (2000)
- ‘After their co-culture with HC-MVECs, SSc BM-MSCs underwent to a phenotypic modulation which re-programs these cells toward a pro-angiogenic behaviour.’ (2013)
- "RUN had significantly (p<0.05) greater size-adjusted CSMI and BSI than C, SWIM, and CYC; and higher size, age, and YST-adjusted CSMI and BSI than SWIM and CYC." (2002)

In this article we report trends in the use of acronyms in the scientific literature from 1950 to the present. We examined acronyms because they can be objectively identified and reflect changes in specialisation and clarity in writing.

## Results

We analysed 24,873,372 titles and 18,249,091 abstracts published between 1950 and 2019, which yielded 1,112,345 unique acronyms. We defined an acronym as a word in which half or more of the characters are upper case letters. For example, mRNA and BRCA1 are both acronyms according to this definition, but N95 is not because two of the three characters are not upper case letters.

**Figure 1.**
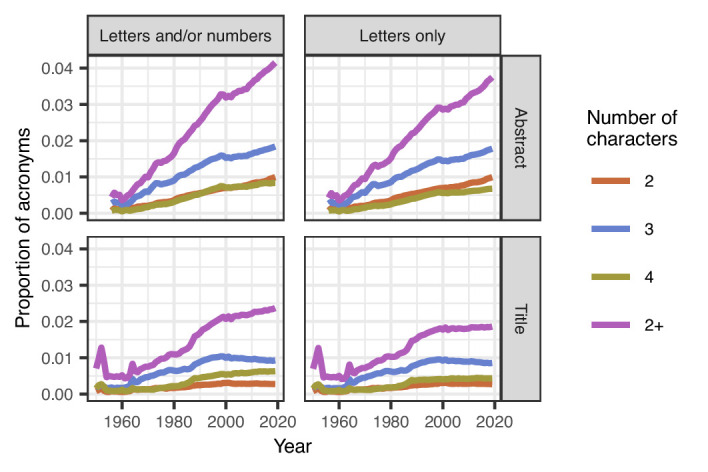
Mean proportions of acronyms in titles and abstracts over time. The proportion of acronyms (purple line) has risen steadily over time in abstracts both for acronyms that are letters and/or numbers (top left) or just letters (top right). Acronyms are generally less common in titles than abstracts, and the proportion in titles has been relatively stable since 2000, but there was an increase from 1960 to 2000 (bottom left and right). Three-character acronyms (blue lines) are more common than two-character acronyms (brown-orange lines) and four-character acronyms (olive green lines) in both titles and abstracts. A sufficient number of abstracts only became available from 1956. The spikes in titles for acronyms of length 2+ in 1952 and 1964 are because of the relatively small number of papers in those years, with over 78,000 papers being excluded in 1964 because the title was in capitals.

We found that the proportion of acronyms in titles increased from 0.7 per 100 words in 1950 to 2.4 per 100 words in 2019 (Figure 1); the proportion of acronyms in abstracts also increased, from 0.4 per 100 words in 1956 to 4.1 per 100 words in 2019. There was at least one acronym in 19% of titles and 73% of abstracts. Three letter acronyms (jokingly called TLAs) were also more popular than acronyms of two and four letters.

**Figure 1—figure supplement 1.**
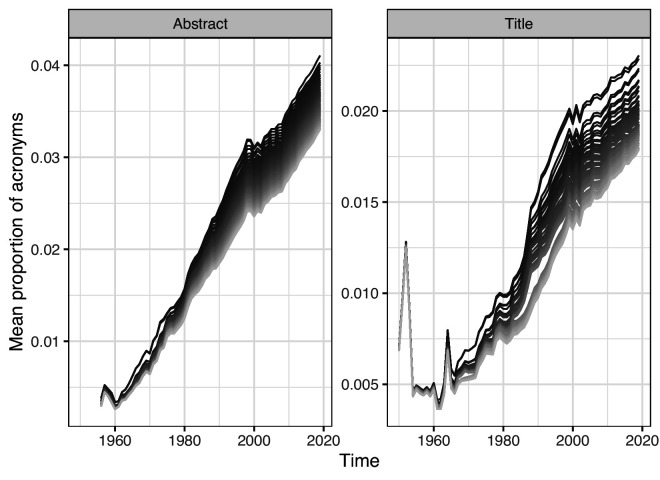
Mean proportions of acronyms in titles and abstracts over time, with the 100 most popular acronyms excluded. Each line shows the trend after excluding up to the *n* most popular acronyms (*n* = 1, ..., 100). The darkest line is for *n* = 1, and the lightest line is for *n* = 100. The number of titles and journals in the early 1950s is much smaller, hence the more erratic trend for titles in that decade.

**Figure 1—figure supplement 2.**
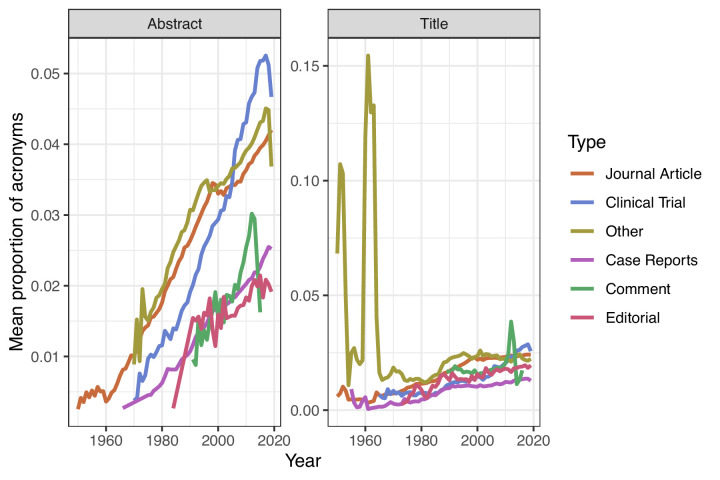
Mean proportions of acronyms in titles and abstracts over time by article type. Data for six article types (journal article, clinical trial, case report, comment, editorial, and other). The high proportion of acronyms in the 1950s and 1960s for ‘other’ is driven by a relatively large number of obituaries that include qualifications, such as FRCP (Fellow of the Royal College of Physicians) or DSO (Distinguished Service Order). The drop in the proportion of acronyms in 2019 for ‘clinical trials’ and ‘other’ may be due to a delay in papers from some journals appearing in PubMed.

**Figure 1—figure supplement 3.**
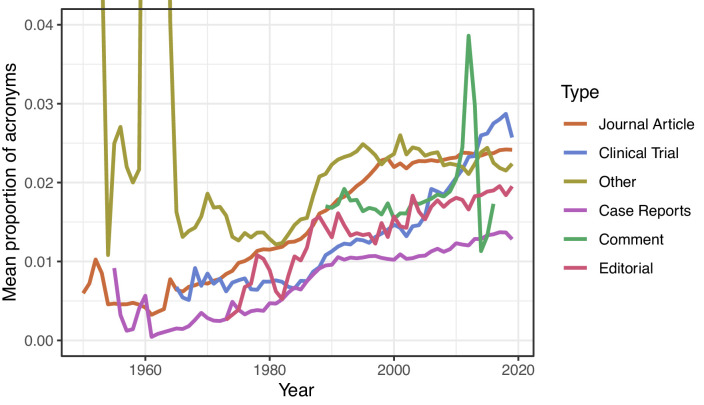
Mean proportions of acronyms in titles over time by article type with a truncated y−axis. Using a truncated y−axis more clearly shows the upward trend in the use of acronyms in titles for all article types over time (by reducing the influence of ‘other’ in the 1950s and 1960s; see Figure 1—figure supplement 2).

The proportion of acronyms in titles has flattened since around the year 2000, whereas the proportion in abstracts continued to increase. Moreover, when the 100 most popular acronyms were removed, there was still a clear increase in acronym use over time (Figure 1—figure supplement 1). Furthermore, the increase was visible in all the article types we studied (including articles, clinical trials, case reports, comments and editorials: Figure 1—figure supplement 2; Figure 1—figure supplement 3). Video 1 shows the top ten acronyms in titles for every year from 1950 to 2019, and Video 2 shows the top ten acronyms in abstracts over the same period.

**Video 1.** The top ten acronyms in titles for every year from 1950 to 2019.

**Video 2.** The top ten acronyms in abstracts for every year from 1950 to 2019.

**Figure 2.**
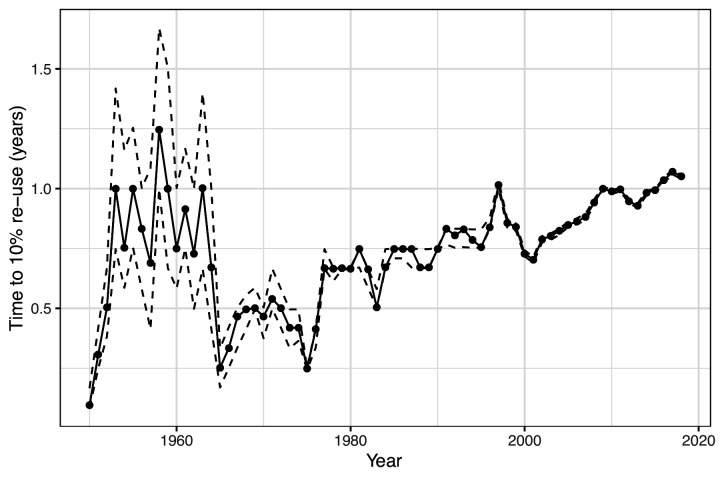
Estimated time to re-use of acronyms over time. The solid line is the estimated time in years for 10% of newly coined acronyms to be re-used in the same journal. 10% was chosen based on the overall percentage of acronyms being re-used within a year. Newly coined acronyms are grouped by year. The dotted lines show the 95% confidence interval for the time to re-use, which narrows over time as the sample size increases. The general trend is of an increasing time to re-use from 1965 onwards, which indicates that acronyms are being re-used less often. The relatively slow times to re-use in the 1950s and early 1960s are likely due to the very different mix of journals in that time.

There are 17,576 possible three-letter acronyms using the upper case letters of the alphabet. We found that 94% of these combinations had been used at least once. Strikingly, out of the 1.1 million acronyms analysed, we found that the majority were rarely used, with 30% occurring only once, and 49% occurring between two and ten time times. Only 0.2% of acronyms (just over 2,000) occurred more than 10,000 times. One year after their first use, only 11% of acronyms had been re-used in a different paper in the same journal. Longer acronyms were less likely to be re-used, with a 17% re-use for two-character acronyms, compared with just 8% for acronyms of five characters or longer. The time taken for acronyms to be re-used has also increased over time (Figure 2), indicating that acronyms created today are less likely to be re-used than previously created acronyms.

DNA is by far the most common acronym and is universally recognised by scientists and the public (Table 1). However, not all the top 20 may be so widely recognised, and it is an interesting individual exercise to test whether you, the reader, recognise them all. Six of the top 20 acronyms also have multiple common meanings in the health and medical literature, such as US and HR‚ although the meaning can usually be inferred from the sentence.

**Table 1.** Top 20 acronyms found in over 24 million titles and over 18 million abstracts. How many do you recognise?

Rank	Acronym	Common meaning(s)	Count
1	DNA	Deoxyribonucleic acid	2,443,760
2	CI	Confidence interval	1,807,878
3	IL	Interleukin/Independent living	1,418,402
4	HIV	Human immunodeficiency virus	1,172,516
5	mRNA	Messenger ribonucleic acid	1,107,547
6	RNA	Ribonucleic acid	1,060,355
7	OR	Odds ratio/Operating room	788,522
8	PCR	Polymerase chain reaction	745,522
9	CT	Computed tomography	743,794
10	ATP	Adenosine triphosphate	582,838
11	MS	Multiple sclerosis/Mass spectrometry	567,523
12	MRI	Magnetic resonance imaging	504,823
13	TNF	Tumour necrosis factor	454,486
14	US	United States/Ultrasound/Urinary system	436,328
15	SD	Standard deviation	411,997
16	NO	Nitric oxide	394,777
17	PD	Parkinson's disease/Peritoneal dialysis	389,566
18	HR	Heart rate/Hazard ratio	383,027
19	IFN	Interferon	383,011
20	CD4	Cluster of differentiation antigen 4	363,502

**Figure 3.**
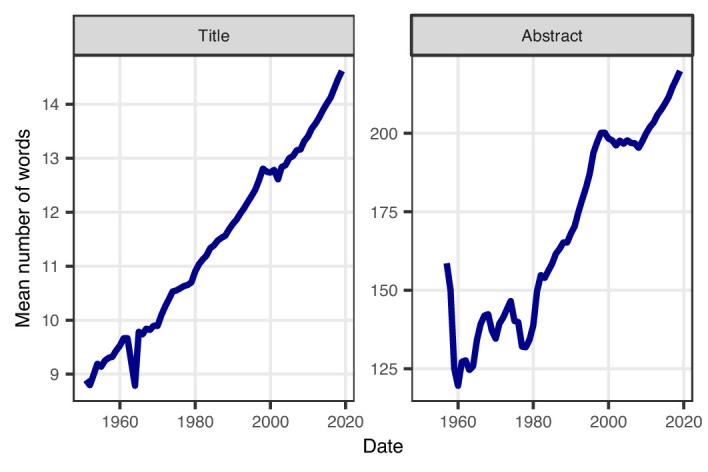
Average number of words in abstracts and titles over time. The average title length has increased linearly between 1950 and 2019 (left). The average length of abstracts has also increased since 1960, except for a brief reduction in the late 1970s and a short period of no change after 2000 (right). A sufficient number of abstracts only became available from 1956. Note that the y-axes in the two panels are different, and that neither starts at zero, because we are interested in the relative trend.

In parallel with increasing acronym use, the average number of words in titles and abstracts has increased over time, with a steady and predominantly linear increase for titles, and a more nonlinear increase for abstracts (Figure 3). The average title length increased from 9.0 words in 1950 to 14.6 words in 2019, and shows no sign of flattening. The average abstract length has also increased, from 128 words in 1962 to 220 words in 2019, and again this trend shows no sign of flattening. It is worth pointing out that these increases have happened despite the word and character limits that many journals place on the length of titles and abstracts.

## Discussion

Our results show a clear increase over time in the use of acronyms titles and abstracts (Figure 1), with most acronyms being used less than 10 times. Titles and abstracts are also getting longer (Figure 3), meaning readers are now required to read more content of greater complexity.

There have been many calls to reduce the use of acronyms and jargon in scientific papers (see, for example, Talk Medicine BMJ, 2019, which recommends a maximum of three acronyms per paper), and many journal and academic writing guides recommend a sparing use of acronyms (Sword, 2012). However, the trends we report suggest that many scientists either ignore these guidelines or simply emulate what has come before. Entrenched writing styles in science are difficult to shift (Doubleday and Connell, 2017), and the creation of new acronyms has become an acceptable part of scientific practice, and for clinical trials is a recognised form of branding (Pottegård et al., 2014).

We believe that scientists should use fewer acronyms when writing scientific papers. In particular, they should avoid using acronyms that might save a small amount of ink but do not save any syllables, such as writing HR instead of heart rate (Pinker, 2015; Lang, 2019). This approach might also make articles easier to read and understand, and even help avoid potential confusion (as HR can also mean hazard ratio or hour). For more complex phrases with multiple syllables and specialist words, such as methylcyclopentadienyl manganese tricarbonyl (MMT), acronyms may ease reading and aid understanding, although MMT might mean methadone maintenance treatment to some readers.

It is difficult to make a general rule about which acronyms to keep and which to spell out. However, there is scope for journals to reduce the use of acronyms by, for example, only permitting the use of certain established acronyms (although the list of allowed acronyms would have to vary from journal to journal). In the future it might be possible, software permitting, for journals to offer two versions of the same paper, one with acronyms and without, so that the reader can select the version they prefer.

Our work shows that new acronyms are too common, and common acronyms are too rare. Reducing acronyms should boost understanding and reduce the gap between the information we produce and the knowledge that we use (Jeschke et al., 2019) ‚ without 'dumbing down' the science. We suggest a second use for DNA: do not abbreviate.

## Materials and methods

We use the word acronym to refer to acronyms (such as AIDS), initialisms (such as WHO) and abbreviations that include capital letters (such as BRCA). We use this broad definition because we were interested in shortened words that a reader is either expected to know or could potentially misunderstand. We did not define acronyms as those defined by the authors using parentheses, because many acronyms were not defined.

We defined an acronym as a word in which half or more of the characters are upper case letters. For example, mRNA is an acronym because it has three upper case letters out of four characters. Characters include numbers, so BRCA1 is also an acronym according to our definition, but N95 is not because two of the three characters are not upper case letters. (It should, however, be noted that N95 is an abbreviation for National Institute for Occupational Safety and Health mask that filters at least 95% of airborne particles). Our definition of an acronym was arrived at using trial and error based on the number of acronyms captured and missed.

We included common acronyms (such as AIDS) because it is difficult to make a simple ruling about what is common and hence well accepted. We instead used a sensitivity analysis that excluded the most common acronyms. We did not include acronyms that have become common words and are generally now not written in upper case letters (such as laser).

### Data extraction

The data were extracted from the PubMed repository of health and medical journals provided by the National Library of Medicine. Data were downloaded between 14 and 22 April 2020. Although the PubMed repository includes papers going back to the year 1781, we used 1950 as the first year. This is because although acronyms have been around for 5,000 years, their use greatly increased after the Second World War (Cannon, 1989) and there were a relatively small number of papers in PubMed prior to 1950. The details of the algorithm to extract acronyms are in Appendix 1.

### Random checks of the algorithm

One researcher (AB) randomly checked 300 of each of the following by hand to verify our acronym-finding algorithm:

- Titles that were excluded
- Titles defined as having no acronyms
- Titles defined as having at least one acronym
- Abstracts defined as having no acronyms
- Abstracts defined as having at least one acronym

The numbers in Table 2 are the count of errors and estimated upper bound for the error percentage using a Bayesian calculation based on the 95th percentile of a beta distribution using the observed number of papers with and without errors. The average error rates were between 0.3% and 6.3%. Zero error rates are unlikely given the great variety of styles across journals and authors. Examples of acronyms missed by our algorithm include those with a relatively large number of lower case letters, numbers, symbols or punctuation (such as circRNA). Examples of acronyms wrongly included by our algorithm include words written in capitals for emphasis and the initials of someone's name appearing in the title as part of a Festschrift.

**Table 2.** Errors made by the algorithm in random samples of titles and abstracts, the number of times that error was made, the average error percentage, and the estimated upper limit.

Error	Count	Average error (%)	Upper limit on error (%)
Wrongly excluded whole title	1	0.3	1.6
Missed valid acronym from title	7	1.2	2.2
Wrongly included acronym from title	5	0.8	1.7
Missed valid acronym from abstract	19	6.3	9.1
Wrongly included acronym from abstract	2	0.7	2.1

### Exclusion reasons and numbers

Table 3 lists the most common reasons why papers were excluded from our analysis. Papers were excluded if they were not written in English, and this was the main exclusion reason for titles. Over 7 million papers had no abstract and around 10,000 had an abstract that was empty or just one word long. Over 298,000 titles and 112,000 abstracts had 60% or more of words in capital letters, making it difficult to distinguish acronyms. This 60% threshold for exclusion was found using trial and error.

**Table 3.** Reasons for excluding titles and abstracts, along with the numbers excluded for each reason.

Reason	Titles	Abstract
No abstract	n/a	7,253,053
Non-English	4,783,569	4,783,569
Pre-1950	384,436	7,973
Title/abstract largely in capitals	298,284	112,369
One word title/abstract	76,303	201
Empty title/abstract	149	9,887
Missing PubMed date	1,510	1,510
Duplicate PubMed ID	1,344	1,328
No article type	109	0
Total excluded	5,545,704	12,169,890
Total included	24,873,372	18,249,091

### Statistical analysis

We examined trends over time by plotting annual averages. The trends were the average number of words in the title and abstract, and the proportion of acronyms in abstracts and titles using word count as the denominator. To examine varied types of acronyms, we split the trends according to acronyms that were letters only compared with those using letters and numbers. We also examined trends according to the length of the acronym. We calculated 95% confidence intervals for the annual means, but in general these were very close to the mean because of the enormous sample size, and hence we do not present them.

We examined the re-use of acronyms after their first use by calculating the number of acronyms re-used in a different paper in the same journal up to one year after their first use. We used the same journal in an attempt to standardise by field and so reduce the probability of counting re-uses for different meanings (such as ED meaning emergency department in the field of emergency medicine and eating disorder in the field of psychology). We counted re-use in the title or abstract. We censored times according to the last issue of the journal. We examined whether re-use was associated with the length of the acronym.

All data management and analyses were made using R version 3.6.1 (R Core Team, 2020).

### Data and code availability

The analysis code and data to replicate all parts of the analyses and generate the figures and tables are available from GitHub: https://github.com/agbarnett/acronyms (Barnett, 2020; copy archived at https://github.com/elifesciences-publications/acronyms). We welcome re-use and the repository is licensed under the terms of the MIT license.

### Limitations

Our algorithm missed a relatively large number of acronyms that included symbols, punctuation and lower case letters. This means our estimates likely underestimate the total number of acronyms. We assumed this error is balanced over time, meaning our trends reflect a real increase in acronym use. We only examined abstracts and titles, not the main text. This is because we used an automated procedure to extract the data, and large numbers of main texts are only available for open access journals and only for recent years, making these data unsuitable for examining broad trends across the scientific literature. We took a broad look at overall trends and did not examine trends within journals or fields. However, our code and data are freely available, and hence other trends and patterns could be examined.

## Data Availability

The analysis code and data to replicate all parts of the analyses and generate the figures and tables are available from GitHub: https://github.com/agbarnett/acronyms (copy archived at https://github.com/elifesciences-publications/acronyms). We welcome re-use and the repository is licensed under the terms of the MIT license. Data was originally downloaded from the PubMed Baseline Repository (March 23, 2020; https://ftp.ncbi.nlm.nih.gov/pubmed/baseline/).

## Additional files

Transparent reporting form

## Appendix 1

### Text processing algorithm

- The data were downloaded in eXtensible Markup Language (XML) format and read into R for processing. The complete R code is freely available on GitHub. The list of key processing steps in order are below. Unless otherwise stated, each step was applied to the title and abstract.
- Remove commonly used copyright sentences such as ‘This article is protected by copyright’ from the abstract.
- Extract the PubMed ID, date the paper was added to PubMed, title, abstract, journal name, article type, language, and number of authors.
- Remove copyright sections and sub-headings from the abstract (such as ‘BACKGROUND’).
- Remove systematic review registrations from the abstract.
- Exclude papers not in English.
- Exclude titles and abstracts that are empty.
- Exclude abstracts of 10 words or fewer that have the article type Published Erratum because they are usually just a citation/note.
- Remove Roman numerals from 1 to 30 (that is, from I to XXX). The upper limit of 30 was based on trial and error. Also remove Roman numerals suffixed with a lower case letter or ‘th’.
- Remove the chromosomes: XX, XY, XO, ZO, XXYY, ZW, ZWW, XXX, XXXX, XXXXX, YYYYY.
- Remove symbols, including mathematical symbols, spacing symbols, punctuation, currencies, trademarks, arrows, superscripts and subscripts.
- Remove common capitals from the title (such as WITHDRAWN, CORRIGENDUM and EDITORIAL).
- Remove sub-headings (such as SETTING and SUBJECTS) from the abstract. (This step is performed in addition to the sub-heading removal step mentioned above).
- Replace common units in the abstract with ‘dummy’ (for example, change ‘kg/m2’ to ‘dummy’) so that they are counted as one word during word counts.
- Remove full-stops in acronyms (so that, for example, W.H.O. becomes WHO).
- Replace all plurals by replacing "s " with a space. This removes plurals from acronyms: for example, EDs becomes ED.
- Break the title and abstract into words.
- Exclude the title or abstract if 60% or more of the words are in capitals as this is a journal style and makes it hard to find acronyms.
- Exclude abstracts with strings of four or more words in capitals. Trial and error showed that this was a journal style and it makes it hard to spot acronyms.
- Exclude the title if the first four words or last four words are in capitals. This is a journal style.
- Remove gene sequences defined as characters of six or more than only contain the letters A, T, C, G, U and p.
- Find and exclude citations to the current paper in the abstract, as these often have author initials which look like acronyms.
- Exclude titles of just one word.
- Exclude papers with a missing PubMed Date.
- Extract acronyms, defined as a word where half or more of the characters are upper case.
- Exclude acronyms longer than 15 characters, as these are often gene strings.

We did not include the following as acronyms:

- Common units of measurement like pH‚ hr or mL.
- Chemical symbols like Na or Ca.
- Acronyms that have become common words (such as laser).
- e.g. and i.e.
